# Cerebral sinovenous thrombosis in a child with ulcerative colitis: A case report

**DOI:** 10.1097/MD.0000000000018649

**Published:** 2020-01-10

**Authors:** Yue Liu, Dongmei Ren, Qiaoyu Zhou, Lin Gao

**Affiliations:** a Department of Neurological Intensive Care Unit; b Department of Gastroenterology, The First Affiliated Hospital of Zhengzhou University, Zhengzhou, Henan, China.

**Keywords:** anticoagulant therapy, cerebral sinovenous thrombosis, inflammatory bowel disease

## Abstract

**Rationale:**

Cerebral sinovenous thrombosis (CVT) associated with inflammatory bowel disease (IBD) is infrequent, but clinically nonnegligible due to its high disability and fatality rates.

**Patient concerns:**

A 12-year-old child with newly developed ulcerative colitis (UC) suffered from a sudden left-sided hemiparesis and numbness.

**Diagnoses:**

Cerebral sinovenous thrombosis due to ulcerative colitis was diagnosed in this girl.

**Interventions:**

The patient was treated with blood transfusion and anticoagulation therapy. Digital subtraction angiography (DSA) and urokinase thrombolysis were implemented followed.

**Outcomes:**

The patient achieved a complete recovery of limb functions and did not present any other stroke recurrences at follow-up a year later.

**Lessons:**

CVT in UC is a serious condition and can occur in the children and adolescents. Rapidly diagnosis of this complication of IBD and apply anticoagulant therapy early can contribute to avoiding a potentially fatal outcome.

## 1 Introduction

Inflammatory bowel diseases (IBDs), a group of chronic systemic inflammatory disease of the gastrointestinal tract, mostly comprise ulcerative colitis (UC) and Crohn disease (CD). It is generally accepted that the disease is a consequence of complex interaction of environmental factors, genetic susceptibility, and microbial influences.^[1]^ These disorders are common enough in children and adolescents that approximately 25% of IBD patients develop the disease before the age of 20.^[2]^

IBD patients have a markedly increased risk of thrombotic complications.^[3]^ For IBD patients, it is considered that 1.3% to 6.4% of adults and 3.3% of children develop cerebrovascular complications during their disease.^[4]^ Higher prevalence of hypercoagulability status during the active phase of IBD has been suggested to be an important culprits.^[5,6]^

Cerebral sinovenous thrombosis (CVT) is an infrequent cause of stroke and most often affects young to middle aged adults. It accounts for a quarter of pediatric stroke and affects 1 of 100,000 children per year approximately.^[7]^ It a rare but well recognized extraintestinal manifestation of IBD that can lead to serious and potentially life-threatening event. Clinically, on account of nonspecific presentation and low incidence, there is a lack of information concerning this complication and its management. Therefore, it is not often readily to recognize that treatment may be delayed or not appropriately treated.

We present a case of a 12-year-old child complicated with extensive CVT from acute onset to complete clinical recovery after aggressive anticoagulation therapy and interventional surgery. Our case report has been approved by the Scientific Research and Clinical Trial Ethics Committee of the First Affiliated Hospital of Zhengzhou University.

## 2 Case report

A 12-year-old girl received treatment in a maternal and child care service center on April 08, 2017 for frequent abdominal pain and diarrhea with a little blood. A diagnosis of bacterial infection was suspected and she was administered oral antibiotics like amoxicillin. Symptoms grew progressively worsen over time. Five days later, she was admitted to the same hospital for repeated fever and headache as well as bloody purulent stools. After treatment with cephalosporin, symptoms improved. Six days after admission, she presented a sudden left-sided hemiparesis and numbness, accompanied by intermittent convulsion. Urgent computed tomography (CT) showed an area of low density in the right frontal lobe. A further magnetic resonance imaging (MRI) showed an abnormal signal in the right frontal and left temporal area. Magnetic resonance venogram (MRV) showed the left venous sinus were not visualize with collateral circulation extensiveness formation. Electrocardiogram showed frequent ventricular premature beat. Abdominal ultrasonography revealed thrombus formation in the superior mesenteric arterial hypoechoic. A diagnosis of cerebral infarction was highly suspected. After reducing intracranial pressure and anticoagulant therapy, no improvement was found with her consciousness and hemiparesis, so she was transferred to our hospital on April 25, 2017.

On admission, she was moderately emaciated, hypotensive, and her body temperature was 37.8 °C. She did not have any vascular risk factor. Her personal and family history was normal besides. Neurological examination revealed hemiplegia with a positive Babinski sign on left side.

Biochemical tests revealed hyperleukocytosis (17.4 × 109/L), moderate anemia (hemoglobin, 88 g/L, hematocrit, 27.8%), hypoalbuminemia (albumin, 30.4 g/L), and elevated levels of C-reactive protein and procalcitonin. A coagulation test revealed an elevation of D-Dimer. Antinuclear antibodies, including anticardiolipin, antinuclear antibody, antismooth muscle antibodies, antineutrophil cytoplasm antibody, and rheumatoid factor were negative. A thrombophilia workup including anticardiolipin, antiphospholipid antibodies, homocysteine, antithrombin was performed and showed normal results.

**Figure 1:**
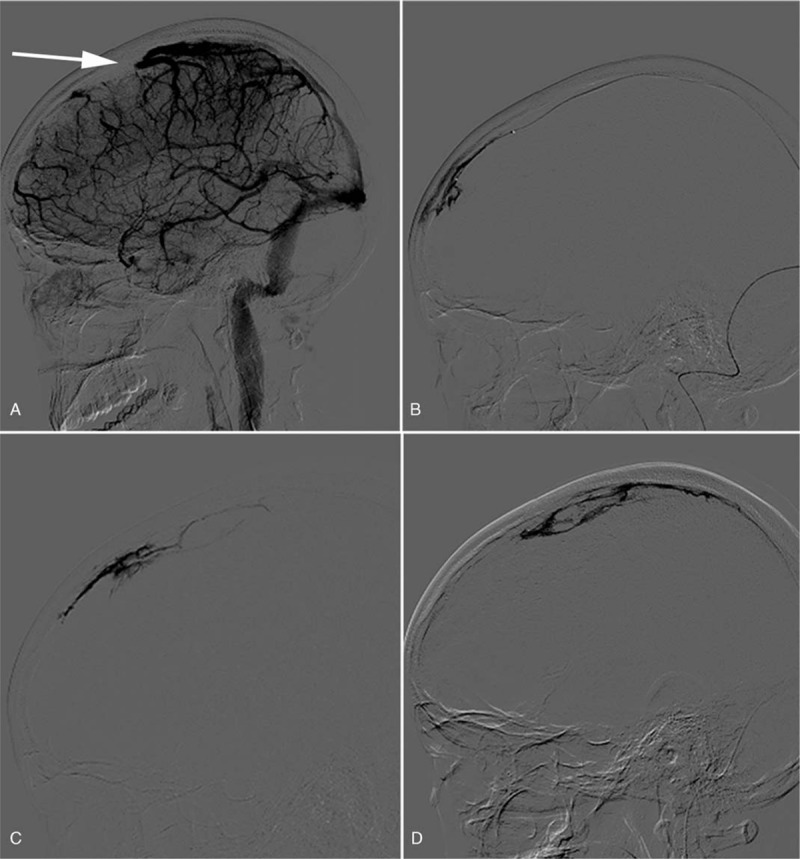
Digital substraction angiography series show vascular occlusion of superior sagittal sinus (A) and the recanalization after therapy (B–D).

**Figure 2:**
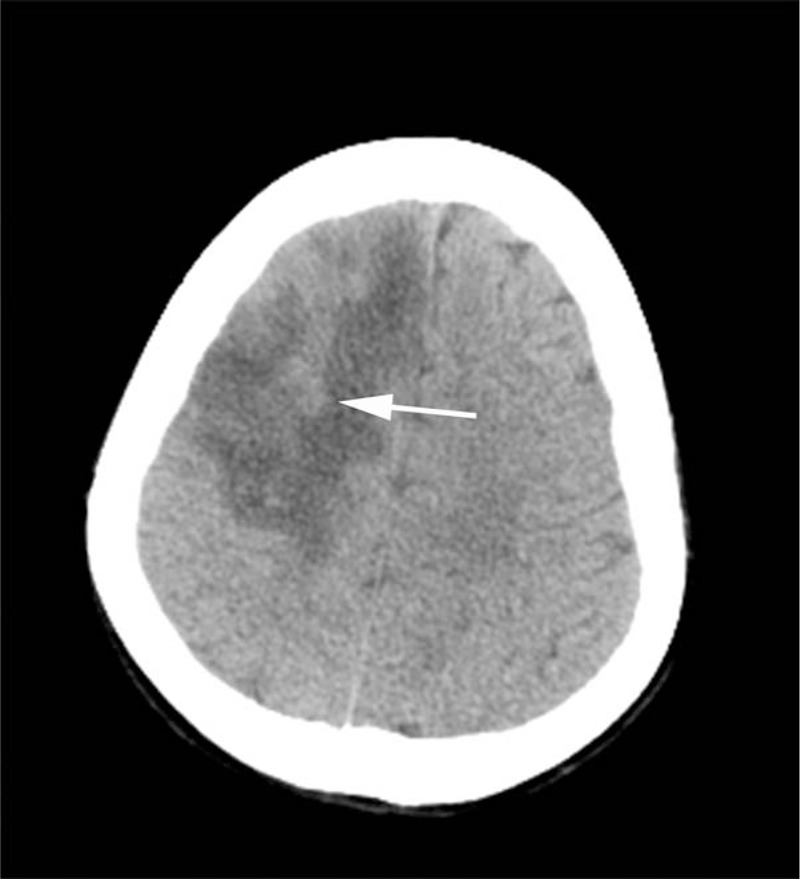
Brain CT at the 10-day follow-up scan shows a massive cerebral infarction in the left frontal lobe. CT = computed tomography.

**Figure 3:**
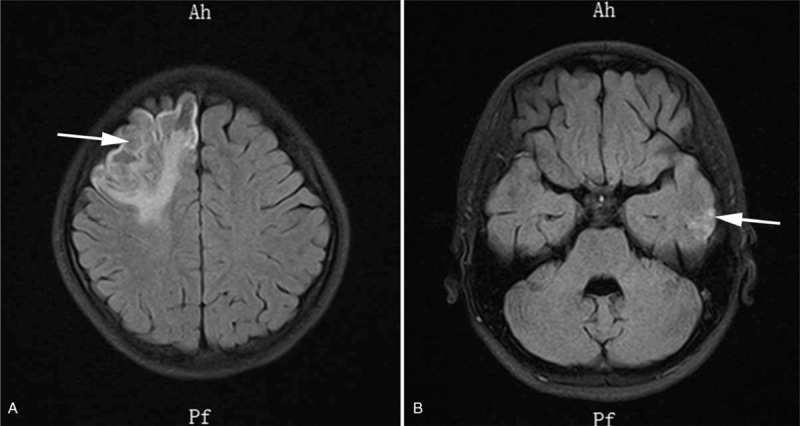
MR venography at the 20-day (A–B) and 6-month (C) follow-up show lack of venous flow through the right transverse and sigmoid sinuses (white arrows).

**Figure 4:**
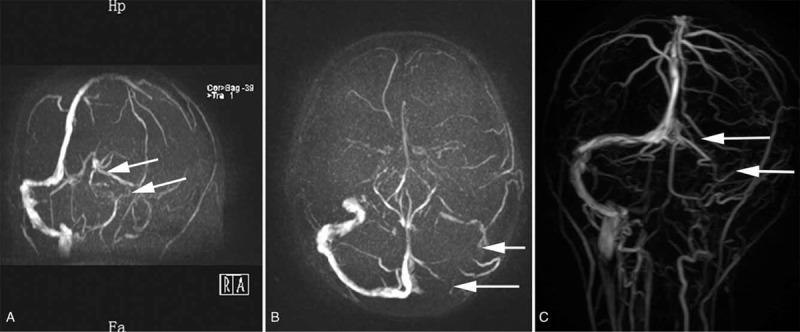
T2-weighted MRI at the 20-day follow-up show residual cerebellar infarctions in the left frontal (A) and temporal (B) lobes (arrows). MRI = magnetic resonance imaging.

After admission, due to severe hematochezia, colonoscopy was unable to perform to determine the cause. At that time the patient was only placed on omeprazole (20 mg QD) for protecting gastrointestinal mucosa. In surprise, symptoms of hematochezia disappeared when treated with frozen plasma and erythrocytes transfusion. The anticoagulation therapy with low-molecular-weight heparin (LMWH; 4000 IU BID) was started, and DSA was performed followed, which confirmed multiple thrombosis of the superior sagittal sinus with secondary hemorrhage (Fig. 1A). Interventional therapy with guide wire was administered simultaneously (Fig. 1B–D). After operation, the patient was treated with Warfarin Sodium Tablets (2.5 mg QD) and LMWH. CT scan (Fig. 2) in 10 days demonstrated a residual left-sided forntal infarction. Twenty days after admission a follow-up MR venography showed partial recanalization of superior sagittal sinus, a filling defect of left sigmoid, and underdevelopment of left transverse sinus (Fig. 3A–B). MRI (Fig. 4) at the same time showed infarctions in the left frontal and temporal lobes. Five days later, with clinical improvement, she was discharged home. She switched to Warfarin Sodium Tablets (2.5 mg QD) only which was withdrawn after a few days with no other recurrent strokes and a slow improvement of her neurological condition.

**Figure 5:**
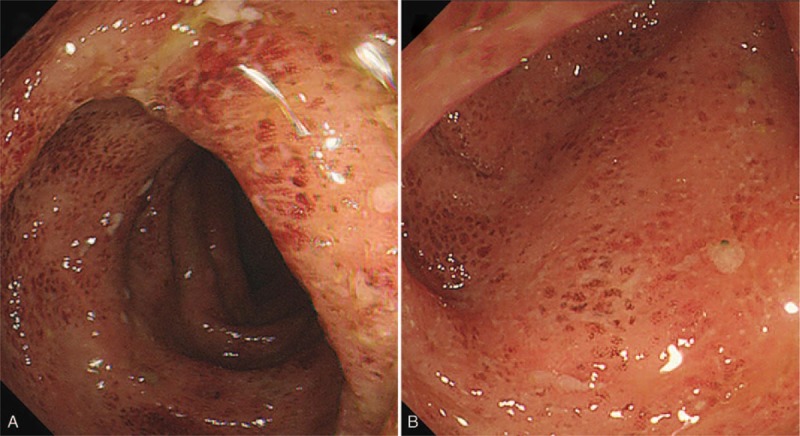
Endoscopic findings show ulcerative colitis (A–B).

The patient received another hospitalization for recurrence after 1 year and a colonoscopy was taken (Fig. 5), then ulcerative colitis was confirmed. A follow-up MR venography showed a similar situation as before (Fig. 3C). At that time she achieved a complete recovery of limb functions and did not present any other stroke recurrences.

## 3 Discussion

CVT that concurrently develops with IBD is rare clinically, and reports in literature in young adults and pediatric patients are much less common. It affects the progression and severity of IBD, with a rising incidence. More than 13% of all patients with CVT are severely disabled and even die.^[8]^ Harrison and Truelove^[9]^ were the first who reported 2 cases of CVT associated with ulcerative colitis in 1967. The youngest patients reported was 7 years old currently.^[10]^ The child we describe here got complete recovery rather than usual stroke recurrences or fatal outcome when compared with the other cases reported in the literature.

There is no authoritative study on the correlation between disease outcome and biochemical features. Specific biomarkers for risk assessment are necessary in diagnosis of CVT in IBD. Elevated fibrinogen level, lipoprotein (a), coagulation factors V, VIII, and IX, membrane procoagulant lipid, Plasminogen Activator Inhibitor-1 (PAI-1), homocysteine; thrombocytosis, and platelet dysfunction have been described during IBD. Deficiency of antithrombin, protein C and S has been most widely investigated. Endothelial protein C receptor, Activated Protein C half-life, protein Z, fibrinolysis, factor XIII, intravascular heparin-like molecules are also decreased.^[11–13]^ Increased D-dimer values on admission was reported to be relative to venous thromboembolism onset and it was taken for an independent predictor associated with venous thromboembolism in hospitalized IBD patients.^[14]^ In the case we reported, markedly increased D-dimer (3.037 mg/L) were observed.

Most patients show prothrombotic risk factors when present. Hereditary factor abnormalities such as Factor V Leiden gene, Methylene tetrahydrofolate reductase gene, Prothrombin G20210A gene, and Factor XIII gene mutation and PAI-1 gene polymorphism have been reported, which are among the main causes of hypercoagulability and hyperhomocysteinemia. Acquired factors are multiple and interrelated, such as hypertension, metabolic syndrome, obesity, pregnancy, smoking, and infection. Systemic inflammation is known to be an effective pro-thrombotic stimulan, the effects of which on hemostatic factors are observed in IBD. They are also age-related that thromboembolism mainly affects patients between the ages of 20 and 30 years. Aside from inflammation, others factors associated with IBD, such as immobilization, dehydration caused by diarrhea, and steroid treatment, are also mentioned.^[15–20]^

The neurological symptoms of CVT in IBD can be extremely variable and there is often a sudden onset. The most common sign at presentation is persistent and mostly global headache.^[21]^ A CVT should be suspected in a patient with IBD who suffer from a recent onset unusual headache. Focal unilateral neurological symptoms are relatively common that tonic clonic seizures and hemiparesis were among early symptoms in our case. Cerebral edema and increased intracranial pressure often leads to vomiting and papilledema. Abnormal facial sensation, mutism, delirium, dizziness, and ataxia have also been reported. In addition, when large lesions are not noticed and no prompt treatment was provided, cerebral herniation and death can occur.^[8,22,23]^

The main controversy of treatment lies in the application of anticoagulant therapy. According to current guidelines for the management of patients with IBD, prophylactic anticoagulation is an effective strategy for preventing vein thrombosis in hospitalized patients with IBD.^[10,24]^ IBD is a prothrombotic condition and microthrombus exists even in the nomal-looking intestinal mucosa. For patients who already have a stroke, anticoagulant therapy is recommended to circumvent thrombus extension and promote spontaneous thrombus resolution.^[23]^ Most researcher suggest to take anticoagulant treatment as soon as the diagnosis is confirmed by imaging techniques despite the presence of a hemorrhagic infarct.^[25]^ However, there is often accompanied by severe intestinal bleeding or secondary intracranial hemorrhage, which may be aggravated by anticoagulant therapy and the risks of further bleeding into the brain tissue outweighed the possible benefits. It is necessary to find a suitable balance point in coagulation and anticoagulation. In addition, oral anticoagulant should be at least 6 months after heparin treatment with a target International normalized ratio of 2.5. But the patient we reported took only a few days of anticoagulant drugs after discharge and achieved a good recovery. Further researches are needed to determine the most effective treatment for this condition.

One issue that has not been addressed in previous case reports is the management of ongoing intestinal bleeding. Generally, the bad intestinal condition is not suitable for a clear diagnosis of colonoscopy. Beyond that, the use of hormones and mesalamine does not relieve symptoms quickly. In the case we present, blood transfusion therapy effectively controls intestinal bleeding. We speculate that this may be related to the marginalization of platelets.

## 4 Conclusions

Cerebral sinus and vein thrombosis is a serious and often fatal complication of idiopathic inflammatory bowel disease if undiagnosed. Awareness of neurological abnormalities is crucial for early and accurate diagnosis of CVT in IBD patients, especially in young people with little or no known cardiovascular risk factors. Despite the rapid progression of massive intracranial infarction, early diagnosis contributes to the use of timely anticoagulant therapy and led to the favorable outcome in our case. In the future, large-scale and multi-center epidemiological, clinical, and pathogenesis research concerning CVT in IBD patients is going be carried out.

## Author contributions

**Data curation:** Yue Liu.

**Project administration:** Lin Gao.

**Software:** Dongmei Ren.

**Supervision:** Lin Gao.

**Validation:** Lin Gao.

**Visualization:** Qiaoyu Zhou.

**Writing – original draft:** Yue Liu, Dongmei Ren.

**Writing – review & editing:** Qiaoyu Zhou, Lin Gao.
